# Short-Term Outcomes of Inguinal Hernia Repair in Older Patients: A Retrospective Review at a Tertiary Center

**DOI:** 10.7759/cureus.18170

**Published:** 2021-09-21

**Authors:** Nouf Akeel

**Affiliations:** 1 Department of Surgery, King Abdulaziz University Faculty of Medicine, Jeddah, SAU

**Keywords:** hernia outcome, short-term outcome, inguinal hernia, elderly people, inguinal hernia repair

## Abstract

Objectives

Although inguinal hernia (IH) repair is low-risk surgery, older patients are occasionally offered watchful waiting because of their functional status and comorbidities. This study reviewed the surgical outcomes of IH repair in older patients in comparison with outcomes in younger patients.

Methods

This retrospective study included all patients who had IH repair from 2010 to 2020. The primary outcomes of interest were postoperative complications and recurrence.

Results

A total of 262 patients underwent IH repair during the study period; 40% were ≥60 years old. One patient had a recurrence. Among the 8% of patients who had postoperative complications, groin pain was the most common one (1.9%). Female patients had a significantly higher rate of complications than male patients did (38.5% female versus 6.4% male, *p*<0.001). The rate of complications was also higher for emergency surgery than for elective surgery (22.6% emergency versus 6.1% elective, *p*<0.001), as well for patients who needed concomitant bowel resection compared with those who did not. Patients who had emergency surgery or postoperative complications had a prolonged hospital stay.

Conclusions

IH repair in older patients is low-risk surgery, comparable to that in younger patients. In this study, emergency surgery was more common in older than in younger patients and posed a higher risk of complications. We recommend offering elective hernia repair to older patients to avoid the higher complication rate associated with emergency repair.

## Introduction

Inguinal hernia (IH) is a common disease among all age groups that requires surgery: 27%-42% of affected male and 3%-6% of affected female patients [1,2]. IH has a high prevalence in the older population (≥65 years), which can be attributed to multiple factors, including abdominal wall weakness, benign prostatic hyperplasia, chronic cough, and chronic constipation, all of which are common in this age group [1-4]. The cumulative probability of pain and irreducibility increases with time (pain 90% and irreducibility 30% at 10 years) [5]. Elective IH repair is considered a safe and low-risk surgery and has a valuable role in preventing hernia-related complications [1,3]. However, older patients are occasionally approached conservatively and denied surgery because of their functional status and the presence of comorbidities [1,4,6-8].

Up to 8% of IH emergency surgery is indicated when incarceration, obstruction, or strangulation occurs as a result of a narrow hernia neck or adhesions between the hernia content and sac [7,9]. Most of those who have emergency surgery are older, and older patients have significantly higher morbidity and mortality rates than younger patients do [2,9].

This study aimed to review the surgical outcomes of IH repair in older patients (≥60 years) and to compare these outcomes with those in a younger age group (<60 years).

## Materials and methods

This retrospective study included all patients who had an elective or emergency IH repair from June 2010 to June 2020 at King Abdulaziz University Hospital. Patients were identified from the operative lists of the electronic health record system. These lists capture all patients with IH who were booked and underwent surgery, as confirmed by operative note details. Patients younger than 18 years or who had a femoral hernia were excluded. Older patients, defined as those who were ≥60 years, were compared with patients who were <60 years. Patient demographics, clinical features, operative details, and postoperative complications were obtained from the electronic medical charts after Institutional Review Board approval (Reference No. 373-21).

The main outcomes of interest included postoperative complications and recurrence at the latest visit to the outpatient clinic. After surgery, patients were followed up during hospitalization, and any adverse events were obtained from inpatient notes. After patients were discharged, follow-up data were retrieved from outpatient visit notes.

Data were analyzed with SPSS version 25 (IBM Corp., Armonk, NY, USA). Qualitative data were expressed as frequencies and percentages. The quantitative data were presented as median or mean and standard deviation (Mean ± SD). The chi-squared test (x^2^) was used to assess the relationship between variables, and the Mann-Whitney and Kruskal-Wallis tests were used for nonparametric variables. Spearman’s correlation analysis and binary logistic regression analysis of the independent predictors of postoperative complications were performed. A two-sided *p*-value of less than 0.05 was considered statistically significant.

## Results

Patient demographics and clinical data

A total of 262 patients underwent IH repair during the study period; 40% were ≥60 years. The mean age of the overall population was 54.28 ± 17.84 years.

Most patients were male (95%) with a mean body mass index (BMI) of 26.73 ± 5.23 kg/m^2^. Older patients were more likely to have comorbid conditions (American Society of Anesthesiologists [ASA] 2: 54%, ASA 3: 87%) compared with their younger counterparts (ASA 2: 43%, ASA 3: 12%). Moreover, a significantly higher percentage of older patients than that of younger patients underwent surgery for recurrent IH (57% older versus 43% younger), and a significantly higher percentage of older patients than that of younger patients underwent emergency surgery (64.5% older versus 35.5% younger, *p*<0.05).

The majority of patients (74%) had an open surgical approach, and around half (54%) had surgery under general anesthesia. Only 12% had an emergency IH repair; three of those patients required concomitant bowel resection (two in the younger group). Most patients (92.7%) received antibiotic prophylaxis, but only 6.9% required thrombosis prophylaxis. Table 1 demonstrates patient, disease, and operative characteristics across both age groups (<60 years versus ≥60 years).

**Table 1:** Relationship between patient age and clinical data (N=262). ASA: American Society of Anesthesiologists. *Mann-Whitney test. †Boldface font indicates statistically significant *p*-values of less than 0.05.

Variable	Age		χ^2^	*p*-value^†^
	<60 years N=157 n (%)	>60 years N=105 n (%)		
ASA classification			57.96	<0.001
ASA 1	102 (82.9)	21 (17.1)		
ASA 2	52 (42.6)	70 (57.4)		
ASA 3	2 (12.5)	14 (87.5)		
ASA 4	1 (100)	0 (0.0)		
Hernia site			0.1	0.751
Bilateral	20 (62.5)	12 (37.5)		
Unilateral	137 (59.6)	93 (40.4)		
Recurrent			4.99	0.025
No	141 (62.7)	84 (37.3)		
Yes	16 (43.2)	21 (56.8)		
Surgical approach			1.5	0.472
Laparoscopic converted to open	2 (50)	2 (50)		
Laparoscopic	43 (66.2)	22 (33.8)		
Open	112 (58)	81 (42)		
Type of anesthesia			0.58	0.745
General	88 (62)	54 (38)		
Local	1 (50)	1 (50)		
Regional	58 (57.6)	50 (42.4)		
Mesh			0.13	0.71
No	6 (54.5)	5 (45.5)		
Yes	151 (60.2)	100 (39.8)		
Emergency surgery			8.74	0.003
No	146 (63.2)	85 (36.8)		
Yes	11 (35.5)	20 (64.5)		
Bilateral or Unilateral repair			0.16	0.685
Bilateral hernia	19 (63.3)	11 (36.7)		
Unilateral hernia	138 (59.5)	94 (40.5)		
Bowel resection			0.89	0.345
No	156 (60.2)	103 (39.8)		
Yes	1 (33.3)	2 (66.7)		
Operation duration (minutes)	105	96	0.59*	0.555
Length of hospitalization (days)	2	2	0.78*	0.436
Follow-up duration (weeks)	2	2	1.09	0.273

The median operative time was 107 minutes, and the median length of hospitalization for the overall population was two days. The median follow-up duration was two weeks. There was no significant difference across groups in terms of bilateral presentation, bilateral repair incidence, or the use of a mesh.

Main outcomes of interest

One patient (0.4%) in the older group had a recurrence during the short follow-up study period. Among the 8% of patients who had postoperative complications, groin pain was the most common one (1.9%). Only 0.8% of patients had postoperative urinary symptoms, and 1.1% had a seroma or hematoma (Table 2).

**Table 2:** Relationship between patient age and postoperative data (N=262).

Variable	Age		χ^2^	*p*-value
	<60 years N=157 n (%)	≥60 years N=105 n (%)		
Groin pain on follow-up			0.84	0.359
No	155 (60.3)	102 (39.7)		
Yes	2 (40)	3 (60)		
Postoperative recurrence			1.5	0.221
No	157 (60.2)	104 (39.8)		
Yes	0 (0.0)	1 (100)		
Overall postoperative complications			2.76	0.096
No	148 (61.4)	93 (38.6)		
Yes	9 (42.9)	12 (57.1)		
Postoperative urinary symptoms			0.08	0.773
No	156 (60)	104 (40)		
Yes	1 (50)	1 (50)		
Postoperative seroma or hematoma			0.89	0.345
No	156 (60.2)	103 (39.8)		
Yes	1 (33.3)	2 (66.7)		

Regarding predictors of postoperative complications (Table 3), there was a trend toward an increased complication rate in the older group compared with the younger group (11.4% older versus 5.7% younger), but it did not reach statistical significance (*p*=0.096). Female patients had a significantly higher rate of complications than male patients did (38.5% female versus 6.4% male, *p*<0.001). The rate of complications was different across groups who received different types of anesthesia, the highest being among those in the general anesthesia group, which was statistically significant. The rate of complications was also higher in the group who underwent emergency IH repair (22.6%) than in those who underwent elective surgery (6.1%), and there was a significant difference in patients who needed concomitant bowel resection versus those who did not. Patients who had emergency surgery or postoperative complications had a prolonged hospital stay, which was statistically significant. The median length of hospitalization was three days for emergency IH surgery, whereas it was two days for elective IH surgery.

**Table 3:** Relationship between postoperative complications and patient characteristics, clinical data, and surgical and postoperative data (N=262). BMI: body mass index, ASA: American Society of Anesthesiologists. *Mann-Whitney test. †Boldface font indicates statistically significant *p*-values of less than 0.05.

Variable	Postoperative complications		χ^2^	*p*-value^†^
	Yes N=21 n (%)	No N=241 n (%)		
Age			2.76	0.096
<60 years	9 (5.7)	148 (94.3)		
≥60 years	12 (11.4)	93 (88.6)		
Gender			17.19	<0.001
Female	5 (38.5)	8 (61.5)		
Male	16 (6.4)	233 (93.6)		
BMI (mean ± SD)	26.62 ± 5.12	26.74 ± 5.25	0.14	0.882
ASA classification			3.56	0.301
ASA 1	7 (5.7)	116 (94.3)		
ASA 2	11 (9)	111 (91)		
ASA 3	3 (18.8)	13 (81.3)		
ASA 4	0 (0.0)	1 (100)		
Hernia site			0.15	0.695
Bilateral	2 (6.3)	30 (93.8)		
Unilateral	19 (8.3)	211 (91.7)		
Recurrent			0.45	0.499
No	17 (7.6)	208 (92.4)		
Yes	4 (10.8)	33 (89.2)		
Surgical approach			1.84	0.397
Laparoscopic converted to open	1 (25)	3 (75)		
Laparoscopic	6 (9.2)	59 (90.8)		
Open	14 (7.3)	179 (92.7)		
Type of anesthesia			6.61	0.0367
General	17 (12)	125 (88)		
Local	0 (0.0)	2 (100)		
Regional	4 (3.4)	114 (96.6)		
Mesh			0.01	0.893
No	1 (9.1)	10 (90.9)		
Yes	20 (8)	231 (92)		
Emergency surgery			10.11	0.001
No	14 (6.1)	217 (93.9)		
Yes	7 (22.6)	24 (77.4)		
Bilateral or Unilateral repair			0.08	0.773
Bilateral hernia	2 (6.7)	28 (93.3)		
Unilateral hernia	19 (8.2)	213 (91.8)		
Bowel resection			34.82	<0.001
No	18 (6.9)	241 (93.1)		
Yes	3 (100)	0 (0.0)		
Groin pain on follow-up			7.07	0.008
No	19 (7.4)	238 (92.6)		
Yes	2 (40)	3 (60)		
Postoperative recurrence			11.52	0.001
No	20 (7.7)	241 (92.3)		
Yes	1 (100)	0 (0.0)		
Postoperative urinary symptoms			23.12	<0.001
No	19 (7.3)	241 (92.7)		
Yes	2 (100)	0 (0.0)		
Postoperative seroma or hematoma			34.82	<0.001
No	18 (6.9)	241 (93.1)		
Yes	3 (100)	0 (0.0)		
Use of antibiotic prophylaxis			0.21	0.646
No	1 (5.3)	18 (94.7)		
Yes	20 (8.2)	223 (91.8)		
Usage of thrombosis prophylaxis			5.42	0.066
No	17 (7)	225 (93)		
Yes	4 (22.2)	14 (77.8)		
Not applicable	0 (0.0)	2 (100)		
Operation duration (minutes)	129	105	1.92*	0.055
Length of hospitalization (days)	3	2	2.79*	0.005
Follow-up duration (weeks)	2	2	0.52	0.601

## Discussion

Older patients are unique in multiple ways, and therefore, physicians approach them differently [4]. IH is a common disease in older individuals, and its repair is associated with low morbidity and mortality rates; however, these patients are occasionally offered watchful waiting [1,4,6-8]. Our study demonstrated that older patients undergoing IH repair have comparable results to those of younger patients with no significant perioperative risk or complications. The overall postoperative morbidity rate was 8%, and the mortality rate was 0%. In the older group, the morbidity rate was 11% versus 6% in the younger patients. Similar findings were published by Rogers and Guzman [8], who found no mortality and no significant difference in postoperative complications between older (>65 years) and younger patients (6% versus 7%). In contrast, a study of more than 29,000 patients from the Danish Hernia Database showed different results, with a significantly higher postoperative morbidity rate in patients ≥65 years (4.5%) than in younger patients (2.7%), as well as a higher 30-day mortality rate (0.12% older versus 0.015% younger) [10]. Although this difference is statistically significant, it does not appear to have clinical significance.

A systematic review assessed the management approach for asymptomatic or mildly symptomatic IH in older male patients (>50 years) [11]. The mean mortality rate in elective surgery was 0.2% and in emergency surgery was 4%. The watchful approach was associated with a mean yearly risk of irreducibility of 0.4% (0.2%-2.7%). Of the patients who were initially managed nonoperatively, 13% eventually required surgery.

Regarding the surgical approach, there was no significant difference between the rates of open versus laparoscopic approaches in either group, and the approach did not affect surgical outcomes. Laparoscopic IH repair is considered a safe and acceptable alternative to open repair, especially for bilateral and recurrent hernias [12]. Hernandez-Rosa et al. [13] demonstrated that laparoscopic IH repair has comparable morbidity and mortality rates to those for open repair in octogenarians. A randomized clinical trial compared Lichtenstein open repair with totally extraperitoneal laparoscopic repair in 1,513 male patients with primary unilateral IH. It demonstrated that the minimally invasive approach was associated with less postoperative pain, shorter sick leave, and faster recovery [14]. In the present study, laparoscopic repair was offered to 25% of patients overall. However, there was no difference in surgical approach between study groups.

Almost two-thirds of the emergency cases in the present study occurred in older patients (64.5%). Three patients had a bowel resection and developed complications, two of which were in the younger group. Complications developed in 22.6 % (7/31) of the overall population who underwent emergency surgery. In addition, emergency surgery was associated with longer hospitalization. Our results concur with several studies that showed that emergency surgery carries higher morbidity and mortality rates, especially in older adults [8,15,16].

We reported a higher operative time (17 ± 47.2 minutes) and length of hospitalization (2.42 ± 3.48 days) than has been reported in the literature [10,12,17,18]. Our hospital admission policy and the fact that it is an educational institution can explain this difference.

Interestingly, the female patients in this cohort had a significantly higher rate of postoperative complications than the male patients did (38% female versus 6% male). Having a higher BMI could explain this association; as in female patients, the rate of obesity (BMI ≥30) was 38% versus 24% in male patients. Köckerling et al. [7] reported a similar correlation for older women who underwent emergency groin surgery. They found that patients who required bowel resection during emergency groin hernia repair and female patients who were ≥66 years had unfavorable perioperative outcomes [7]. Another study by Nilsson et al. [9] showed that female patients had a greater risk of postoperative mortality, which can be attributed to a higher rate of emergency procedures irrespective of the type of hernia.

This study has several limitations. It was a retrospective design, and the overall number of older patients was small. Given the small number and low complication rates, other predictors of postoperative complications or differences in outcomes in the older compared with the younger population may not be detected. Moreover, there was no standardized postoperative pathway or designated follow-up to formally assess patient outcomes prospectively. Lastly, the follow-up time was short and not standardized for all patients.

## Conclusions

The risk of morbidity associated with IH repair is low in older patients, comparable to that in younger patients. Therefore, we recommend offering elective hernia repair to older patients to avoid the higher complication rates associated with an emergency repair. Local data that evaluate IH repair in older compared with younger patients are scarce. Prospective studies will add to our knowledge about IH repair in older adults.

## References

[1] Ceresoli M, Carissimi F, Nigro A, Fransvea P, Lepre L, Braga M, Costa G (2020). Emergency hernia repair in the elderly: multivariate analysis of morbidity and mortality from an Italian registry [PREPRINT]. Hernia.

[2] The HerniaSurge Group (2018). International guidelines for groin hernia management. Hernia.

[3] Li L, Pang Y, Wang Y, Li Q, Meng X (2020). Comparison of spinal anesthesia and general anesthesia in inguinal hernia repair in adult: a systematic review and meta-analysis. BMC Anesthesiol.

[4] Tonelli C, Ringhouse B, Bunn C, Luchette F (2021). The impact of the aging population on surgical diseases. Curr Geriatr Rep.

[5] Hair A, Paterson C, Wright D, Baxter JN, O’Dwyer PJ (2001). What effect does the duration of an inguinal hernia have on patient symptoms?. J Am Coll Surg.

[6] Chlebny T, Zelga P, Pryt M, Zelga M, Dziki A (2017). Safe and uncomplicated inguinal hernia surgery in the elderly - message from anesthesiologists to general surgeons. Pol Przegl Chir.

[7] Köckerling F, Heine T, Adolf D (2021). Trends in emergent groin hernia repair-an analysis from the Herniamed Registry. Front Surg.

[8] Rogers FB, Guzman EA (2011). Inguinal hernia repair in a community setting: implications for the elderly. Hernia.

[9] Nilsson H, Stylianidis G, Haapamäki M, Nilsson E, Nordin P (2007). Mortality after groin hernia surgery. Ann Surg.

[10] Bay-Nielsen M, Kehlet H (2008). Anaesthesia and post-operative morbidity after elective groin hernia repair: a nation-wide study. Acta Anaesthesiol Scand.

[11] INCA Trialists Collaboration (2011). Operation compared with watchful waiting in elderly male inguinal hernia patients: a review and data analysis. J Am Coll Surg.

[12] Hope WW, Bools L, Menon A, Scott CM 3rd, Adams A, Hooks WB 3rd (2013). Comparing laparoscopic and open inguinal hernia repair in octogenarians. Hernia.

[13] Hernandez-Rosa J, Lo CC, Choi JJ, Colon MJ, Boudourakis L, Telem DA, Divino CM (2011). Laparoscopic versus open inguinal hernia repair in octogenarians. Hernia.

[14] Eklund A, Rudberg C, Smedberg S, Enander LK, Leijonmarck CE, Osterberg J, Montgomery A (2006). Short-term results of a randomized clinical trial comparing Lichtenstein open repair with totally extraperitoneal laparoscopic inguinal hernia repair. Br J Surg.

[15] Kulah B, Duzgun AP, Moran M, Kulacoglu IH, Ozmen MM, Coskun F (2001). Emergency hernia repairs in elderly patients. Am J Surg.

[16] Wu JJ, Baldwin BC, Goldwater E, Counihan TC (2017). Should we perform elective inguinal hernia repair in the elderly?. Hernia.

[17] Aydin M, Fikatas P, Denecke C, Pratschke J, Raakow J (2021). Cost analysis of inguinal hernia repair: the influence of clinical and hernia-specific factors [PREPRINT]. Hernia.

[18] Hada G, Zhang S, Song Y, Jaiswar M, Xie Y, Jian F, Lei W (2021). Safety of inguinal hernia repair in the elderly with perioperative continuation of antithrombotic therapy. Visc Med.

